# Tissue-specific mutagenicity of *N*-nitroso-methylphenylamine assayed by the transgenic rodent assay and duplex sequencing

**DOI:** 10.3389/ftox.2026.1994796

**Published:** 2026-10-27

**Authors:** Li Xia, Jian Yan, Kamela Mitchell, Robert H. Heflich, Ying Chen, Timothy J. McGovern, Aisar H. Atrakchi, Tao Chen

**Affiliations:** 1 Division of Genetic and Molecular Toxicology, National Center for Toxicological Research, US Food and Drug Administration, Jefferson, AR, United States; 2 Office of New Drugs, Center for Drug Evaluation and Research, US Food and Drug Administration, Silver Spring, MD, United States

**Keywords:** *cII* mutation assay, duplex sequencing, esophagus mutagenesis and carcinogenesis, mutation signature, *N*-methyl-*N*-nitroso-aniline, *N*-nitroso-methylphenylamine

## Abstract

*N*-Nitroso-methylphenylamine (NMPA) is a rat esophageal carcinogen that has been found in food, tobacco and drugs. In this study, NMPA was administered via drinking water to Big Blue transgenic rats over 28 days following methods recommended in OECD TG 488; benzo[*a*]pyrene (B[a]P) was administered as the positive control. The esophagus, liver, and bone marrow were collected and analyzed for mutation using both the *cII* mutation assay and Duplex Sequencing (DS). NMPA treatment induced tissue-specific mutations, with the esophagus exhibiting the highest mutagenic response in both the *cII* and DS assays, followed by the liver; bone marrow remained relatively unaffected, showing no increase in the *cII* assay and only a slight elevation when analyzed by DS. The DS mutational spectrum for NMPA was characterized by a broadly distributed elevation across all base substitution types and in all trinucleotide contexts, distinguishing it from other small-molecule nitrosamines. Notably, DS detected fold-increases in NMPA mutagenesis greater than those detected by the transgenic rodent (TGR) assay, with a maximum 7.5-fold increase in genomic mutation frequency in the esophagus compared to the 5.6-fold increase in the *cII* gene. These data suggest that NMPA is an organ-specific mutagen, and that its mutagenic potency corresponds with its carcinogenicity in the primary target tissue and other tissues.

## 1 Introduction

*N*-Nitrosamines (or nitrosamines) are formed through reaction between nitrites and amines under acidic or high-temperature conditions; they have been found in a variety of consumer products, including food, tobacco products, and pharmaceuticals (Bharate, 2021; Gushgari and Halden, 2018). Nitrosamines, specifically small-molecule *N*-nitrosamines, are also among the most potent and broadly acting carcinogens, with only 18% of nitrosamines classified as non-carcinogenic (Thresher et al., 2020). Nitrosamines display a strong correlation between mutagenicity and carcinogenicity, implying that their carcinogenicity is principally due to their mutagenicity. However, most nitrosamines require metabolic activation by cytochrome P450 enzymes (CYPs) to exert their carcinogenic effects (Kamataki et al., 2002). Although there is a general qualitative correlation between mutagenicity in bacterial systems and carcinogenicity in animals, a few carcinogenic nitrosamines display weak or no mutagenic effects in standard bacterial assays (Heflich et al., 2024; Lijinsky, 1987a).

Several small-molecule nitrosamines, such as *N*-nitroso-dimethylamine (NDMA), *N*-nitroso-diethylamine (NDEA), *N-*nitroso-methylphenylamine (NMPA), *N*-nitroso-diisopropylamine (NDIPA), *N*-nitroso-isophenylethylamine (NEIPA), *N*-nitroso-dibutylamine (NDBA), and *N*-nitroso-*N*-methyl-4-aminobutyric acid (NMBA), have been singled out as potential impurities in drug products (Finotti Cordeiro et al., 2024). Since 2018, with the detection of NDMA and NDEA as impurities in pharmaceuticals, nitrosamine impurities have raised major regulatory and public health concerns, leading to drug recalls and stricter global regulations (Charoo et al., 2019).

Except for a few well-characterized nitrosamines, like NDMA and NDEA, the toxicity of many structurally diverse and potentially hazardous nitrosamines has not been thoroughly investigated. Among less well-studied nitrosamines, NMPA is a nonvolatile and highly lipophilic nitrosamine that occurs in food and has been detected in pharmaceutical products. It is metabolized by CYPs through α-hydroxylation, producing formaldehyde and a benzene diazonium ion (BDI) – a reactive intermediate that can form DNA adducts like N6-adenine modifications (Carlsson et al., 2025; EFSA Panel on Contaminants in the Food Chain (EFSA CONTAM Panel), 2023; Snodin et al., 2024). However, data on the formation, stability, repair, and *in vivo* mutagenic potential of these adducts are limited. In addition, while many nitrosamines are liver carcinogens, others, including NMPA, display strong carcinogenic effects in other tissues (Lijinsky, 1987b). Given its unique structure characterized by an aromatic amine structure, distinct from simple aliphatic nitrosamines like NDMA, and its widespread presence, further investigation is needed to better understand the mutagenicity, underlying mechanisms, and carcinogenic potential of NMPA.

Previous studies found that NMPA induces tumors primarily in rat esophagus, with additional tumor formation observed in the pharynx and forestomach, and in the lungs of mice. Additionally, the target-organ specificity appears to be independent of the route of administration, with carcinogenic effects observed following exposure via drinking water, intraperitoneal injection, or oral gavage (Boyland et al., 1964; Druckrey et al., 1967; Goodall et al., 1970; Kroeger-Koepke et al., 1983; Schmähl, 1975). Reported TD_50_ values for NMPA are 0.335 mg/kg/day (Lhasa Carcinogenicity Database [CPDB]) and 0.142 mg/kg/day (The Carcinogenic Potency Project) (Gold, 2026; Lhasa, 2026). Its TD_50_ value is only slightly higher than that of NDMA (0.0959 mg/kg/day, CPDB), an example of a Cohort of Concern nitrosamine drug impurity. According to recent studies, NMPA has weak to no effect in the Ames test using rat liver S9; but positive results were observed when hamster liver S9 was used (Heflich, et al., 2024). Mutagenic and genotoxic effects have been evaluated in several *in vitro* studies using yeast, TK6 cells, and HepaRG cells (Kushida et al., 2000; Li et al., 2022a; Li and Hecht, 2022; Seo et al., 2023; Seo et al., 2025).

Under current regulatory recommendations, short-term genotoxicity tests are used to help identify chemical carcinogens as part of a comprehensive assessment strategy. Standard assays, such as the Ames test and *in vitro* mammalian cell gene mutation assays, are commonly included in a genotoxicity test battery (ICH, 2012). While these assays provide valuable insights into a compound’s genetic effects, they are not always predictive of *in vivo* carcinogenic outcomes, especially for compounds, like nitrosamines, that require metabolic activation. Therefore, negative results in standard *in vitro* assays do not always indicate a lack of carcinogenic potential (Singh et al., 2024). To address these limitations, *in vivo* assays, such as the transgenic rodent (TGR) gene mutation assay (OECD, 2025), are used to provide more physiologically relevant data (ICH, 2023). This approach is important for compounds like NMPA, where traditional *in vitro* assays may underestimate genotoxic risk due to species-specific differences (Choudhary et al., 2025).

The TGR assay detects gene mutations in virtually any tissue of transgenic rat and mouse models, with the mutational responses strongly correlated with long-term rodent carcinogenicity data, making the assay a key tool in mutagenicity and carcinogenicity assessment (Lambert et al., 2005). Among TGR models, the Big Blue system employs a lambda shuttle vector containing the *cII* reporter gene. Following exposure, DNA is extracted from different tissues, and the *cII* gene is recovered via phage packaging and quantified by infection of indicator bacteria to determine mutant frequency. Besides being an indicator of mutagenic hazard, the results can be used to compare the relative potency of test compounds compared to well-characterized carcinogens, helping to rank their hazard potential and support regulatory toxicology decisions.

However, currently there is restricted capacity for performing the TGR assay, which is both labor- and resource-intensive (Eastmond et al., 2013; Powley et al., 2024). Another novel technique–Duplex Sequencing (DS), a form of error-corrected next-generation sequencing (ECS), enables detection of low-frequency mutations in genomic DNA. Unlike traditional methods, DS compares sequence reads from both strands of a DNA molecule, dramatically reducing sequencing errors and resulting in the detection of true mutations occurring at frequencies as low as 10^−8^ (Bercu et al., 2023; Dodge et al., 2023; Kennedy et al., 2014; Sahib et al., 2024; Salk et al., 2018; Schmitt et al., 2012). It can be applied to any tissue in transgenic or non-transgenic animals, requiring only small quantities of genomic DNA. In addition to measuring mutation frequency, DS also provides detailed mutation spectra, offering insight into mutagenic mechanisms.

The mutagenicity, potent carcinogenicity, tissue-specificity, and unusual metabolic behavior of NMPA raise significant questions for regulatory toxicology. In this study, NMPA was administered to Big Blue rats for 28 days as recommended by the OECD TG 488 guideline. The liver, esophagus, and bone marrow were collected and both the *cII* assay and DS analysis were conducted in order to make a side-by-side comparison of mutagenic responses across tissues and assay platforms. The tissues were selected for their biological relevance in understanding the carcinogenicity of NMPA. The liver, a primary site of metabolic activation and possessing high levels of CYPs, is a common target for nitrosamines, including NDMA and NDEA (EFSA Panel on Contaminants in the Food Chain (EFSA CONTAM Panel), 2023). The esophagus is the main target tissue for NMPA-induced carcinogenesis, as demonstrated in the rodent studies cited above. Bone marrow was included as a potential surrogate tissue that is used in OECD-supported genotoxicity assays such as the *Pig-a* gene mutation assay and micronucleus assay. If NMPA-induced mutagenicity can be observed using these assays, bone marrow could serve as a practical target tissue for future screening and regulatory assessment. Overall, this study highlights tissue-specific differences and assay sensitivities for evaluating the genotoxic risk of structurally and biologically atypical nitrosamines.

## 2 Materials and methods

### 2.1 Chemicals and animals

*N*-nitroso-methylphenylamine (NMPA; CAS No. 614-00-6) was obtained from TCI America (Portland, OR) and dissolved in water to prepare treatment solutions. Benzo[*a*]pyrene (B[a]P; CAS No. 50-32-8), used as a positive control, was purchased from Sigma-Aldrich (St. Louis, MO) and dissolved in olive oil. Six-week-old male Big Blue rats (Gentronix, Alderley Edge, United Kingdom) were housed at 24 °C under a 12-h light/dark cycle, with access to water and NIH-41 irradiated pelleted diet *ad libitum*. Following a 1-week acclimation, the rats were randomly assigned to treatment groups, with three rats per cage. All experimental procedures were reviewed and approved by the Institutional Animal Care and Use Committee (IACUC) of the National Center for Toxicological Research (NCTR).

### 2.2 Animal treatment and tissue collection

Male Big Blue rats were administered NMPA through drinking water for 28 consecutive days at doses targeted to yield 0, 5, 10, and 20 mg/kg NMPA body weight per day; the doses were based on a previous tumorigenesis study (Kroeger-Koepke, et al., 1983). The concentrations of NMPA solutions were adjusted daily using measurements of water intake and animal weight to yield the targeted doses. The positive control group received 75 mg/kg/day B[a]P via oral gavage for the same duration. The negative control and each NMPA dose group contained six rats; the B[a]P treatment group contained three rats. Animals were sacrificed 3 days after the final treatment. Whole tissues, including whole bone marrow, liver, and esophagus, were collected, flash-frozen in liquid nitrogen without further separation of cell types, and stored at −80 °C until further analysis.

### 2.3 Transgenic *cII* gene mutation assay

Genomic DNA was isolated from liver, esophagus, and bone marrow tissues using the RecoverEase DNA Isolation Kit (Agilent, Santa Clara, CA) according to the manufacturer’s protocol. Lambda shuttle vectors containing the *cII* reporter gene were rescued from the genomic DNA and packaged into infectious lambda phage particles using Transpack packaging extract (Agilent). The resulting phages were used to infect *Escherichia coli* G1250, which were subsequently incubated under selective (24 °C) and non-selective (37 °C) conditions. For total titer determination, infected cultures were plated on TB1 agar plates and incubated at 37 °C for 16–18 h. For mutant selection, cultures were plated on selective TB1 plates and incubated at 24 °C for 40–48 h. Each sample was analyzed using three plates for titer determination and five plates for mutant selection. At least 3 × 10^5^ plaque-forming units (pfus) were evaluated per sample to ensure statistical robustness. The *cII* mutant frequency was calculated by dividing the number of mutant plaques by the total number of plaques counted.

### 2.4 Duplex sequencing (DS) for mutation detection

Genomic DNA previously extracted from liver, esophagus, and bone marrow was used for DS analysis. DNA concentrations were quantified using the Qubit dsDNA Genomic Assay Kit (Thermo Fisher Scientific, Waltham, MA), and DNA quality was assessed using the Genomic DNA ScreenTape Assay (Agilent). All samples had concentrations greater than 50 ng/μL and DNA integrity number (DIN) values above 7. Approximately 1,000 ng of DNA per sample were enzymatically fragmented to a median insert size of ∼250–300 base pairs using the TwinStrand DuplexSeq Enzymatic Fragmentation Module (v1.0). Library preparation was performed according to the TwinStrand DuplexSeq Kit Manual (v1.1), including end-repair, A-tailing, and ligation of DuplexSeq adapters containing unique molecular identifiers (UMIs). Adapter-ligated DNA was PCR-amplified and enriched using the Rat Mutagenesis Panel (TwinStrand Biosciences, Seattle, WA, now available at Scantox, Ejby, Denmark) which targets 48 kb across 20 representative genomic regions with diverse sequence contexts (genic/nongenic, coding/noncoding, and varying GC content). PCR was performed in three steps with 10, 14, and 6 cycles, respectively. Final libraries were quantified using the Qubit dsDNA HS Assay Kit and Agilent TapeStation, pooled, and sequenced on the Illumina NovaSeq 6000 platform with 2 × 150 bp paired end reads. FASTQ files (containing data from ∼1.8 × 10^−8^ reads for each sample) were processed using the TwinStrand DuplexSeq Mutagenesis App on the DNAnexus platform (v4.2.0). The pipeline included UMI-based read grouping, duplex consensus calling, read trimming, decontamination, and variant calling with VarDictJava. Output metrics included mutation frequency (calculated by counting all identical mutations as one independent mutation within a sample), mutational spectra, and trinucleotide context, following established protocols (LeBlanc et al., 2022; Sahib, et al., 2024; Valentine et al., 2020). The number of duplex bases for every sample (∼900 million per sample) met the minimum threshold of 500 million for the data analysis to identify mutations.

### 2.5 Statistical analysis

Body weight relative to the initial treatment day, *cII* gene mutant frequency, and DS data are presented as mean ± S.E.M. Statistical analyses were conducted in SigmaPlot (version 14.5; Systat/Grafiti, Palo Alto, CA). Group differences across treatment conditions in liver, esophagus, and bone marrow were assessed using one-way ANOVA followed by the Holm–Sidak *post hoc* test. A *p*-value <0.05 was considered statistically significant. Mutation frequencies and spectra were analyzed and compared between the treatment and control groups using computational tools. Significant differences between mutational spectra of different treatments groups were analyzed according to a method for mutation spectrum analysis (Cariello et al., 1994).

## 3 Results

### 3.1 Animal body weights

Rats were randomly assigned to receive either vehicle, the positive control B[a]P (via gavage in olive oil), or NMPA (via drinking water). Body weight was monitored to assess systemic toxicity, and relative weight changes over time are shown in Figure 1. Although all animals gained weight during the treatment period, the weight gain in the NMPA- and B[a]P-treated groups was consistently lower than that in the vehicle controls. At the end of treatment and on the day of sacrifice (3 days post-treatment), all treatment groups had statistically significant reductions in body weight (about 10%–15%) compared to the vehicle control. No mortality or severe clinical signs were observed, and the highest dose tested in this study was below the maximum tolerated dose (MTD). Therefore, all doses were considered appropriate for inclusion in the mutagenicity evaluation of this study.

**FIGURE 1:**
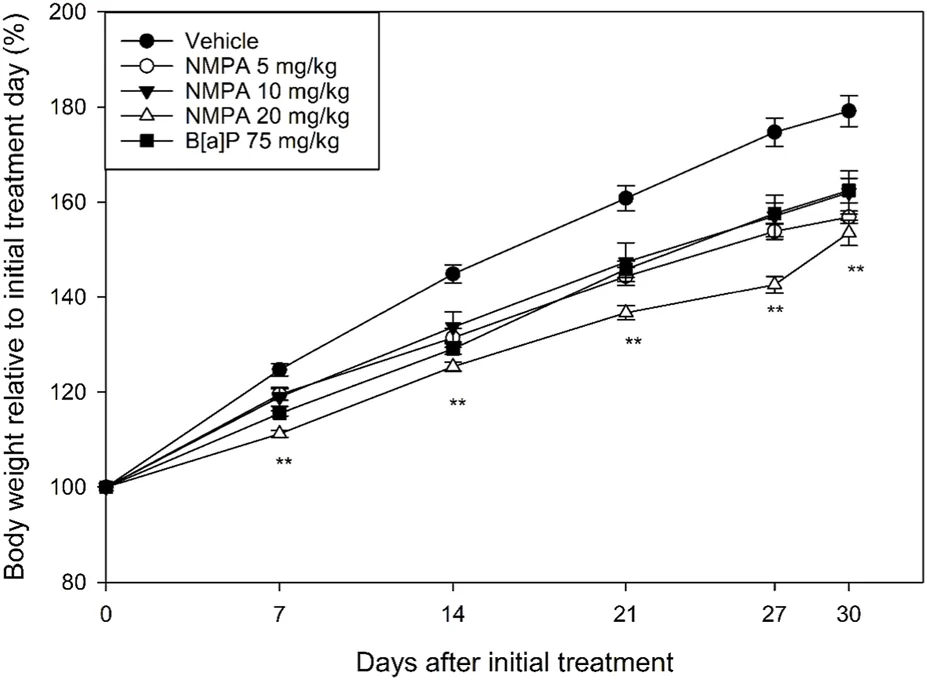
Body weight changes in Big Blue rats during *N*-nitroso-methylphenylamine (NMPA) treatment. Each data point represents the mean ± SEM (n = 6 per group), except for the benzo[*a*]pyrene (B[a]P) positive control group (n = 3). All treatment groups are statistically different from the vehicle control group at all time points after the treatment (**p < 0.05).

### 3.2 Mutant frequency in the *cII* gene

*cII* gene mutant frequencies were assessed in the liver, esophagus, and bone marrow of Big Blue rats using the TGR assay. NMPA-induced mutations were observed in both the liver and esophagus, though no clear dose-dependent response was observed in either tissue (Figure 2A). The esophagus exhibited the greatest increase in mutant frequency among all tissues. In contrast, no significant changes were detected in the bone marrow following NMPA exposure. All three tissues exhibited increased mutant frequencies in the B[a]P-treated group, with liver showing the greatest increase.

**FIGURE 2:**
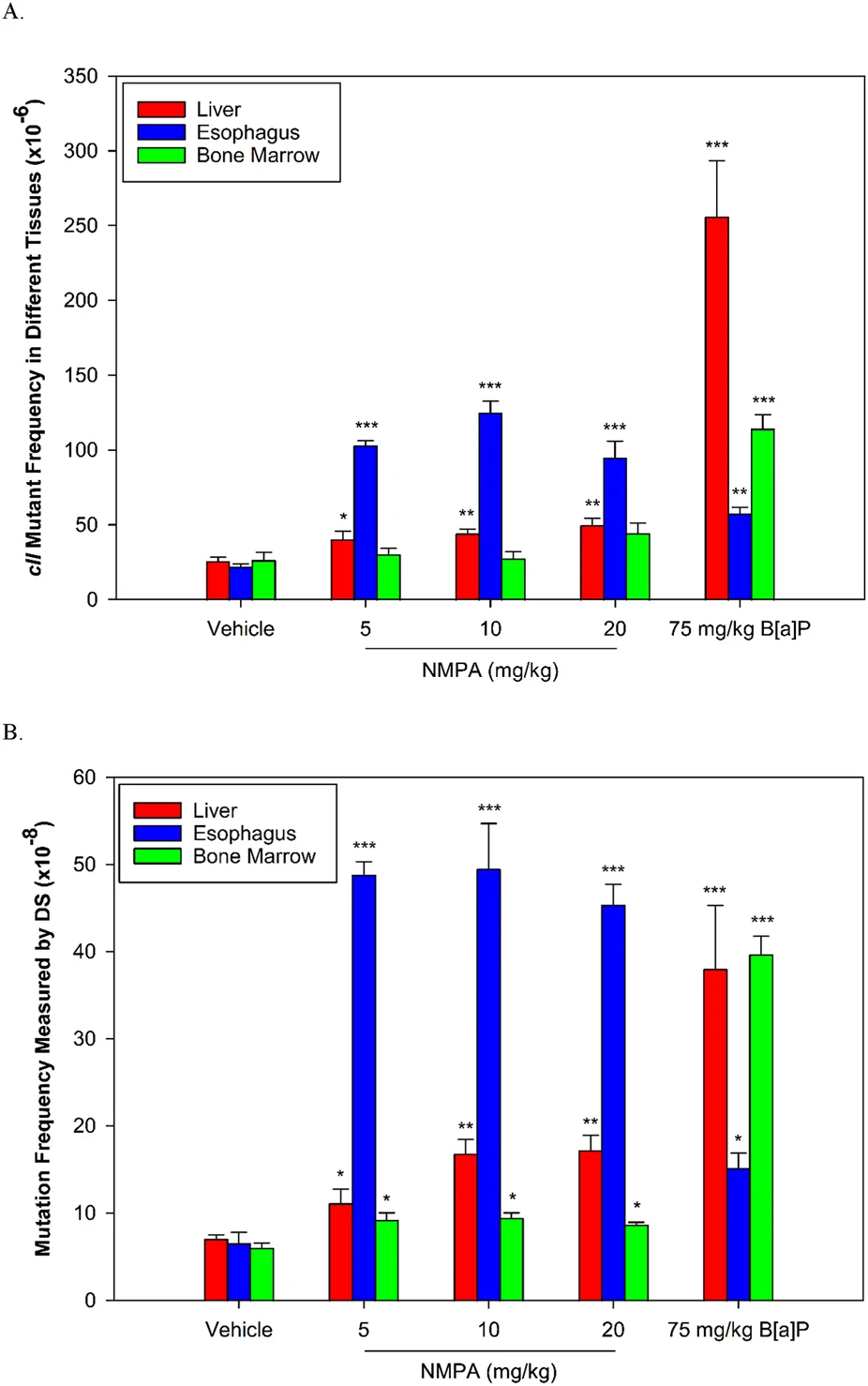
Mutant and mutation frequencies in Big Blue rats treated with vehicle, *N*-nitroso-methylphenylamine (NMPA; 5, 10, or 20 mg/kg), or benzo[a]pyrene (B[a]P; 75 mg/kg). **(A)** *cII* mutant frequencies in the liver, esophagus, and bone marrow measured by the transgenic mutation assay. **(B)** Genomic DNA mutation frequencies in the liver, esophagus, and bone marrow assessed by Duplex Sequencing (DS). Data are presented as mean ± SEM (n = 6 per group), except for the B[a]P group (n = 3). Statistical significance is indicated as *p < 0.05, **p < 0.01, and ***p < 0.001 versus vehicle control.

In the esophagus, the *cII* mutant frequencies for the 0, 5, 10, and 20 mg/kg NMPA-, and B[a]P-treatment groups were 22.2, 103.1, 125, 94.8, and 57.8 × 10^−6^, respectively (Figure 2A); the three NMPA doses generated 4.7-, 5.6-, and 4.3-fold increases compared to the control, while B[a]P produced a 2.6-fold increase. In the liver, the *cII* mutant frequencies were 25.8, 40.5, 44.3, 49.8, and 255.7 × 10^−6^ across the same groups, corresponding to 1.6-, 1.7-, and 1.9-fold increases for the NMPA-treated groups, and a 9.9-fold increase for B[*a*]P. Rat bone marrow did not show a clear mutagenic response to NMPA, with mutant frequencies of 26.6, 30.6, 27.8, and 44.6 × 10^−6^ in the vehicle control and the 3 NMPA-treated groups, respectively (1.1-, 1.0-, and 1.7-fold increases). In contrast, B[a]P led to a 4.3-fold increase in mutant frequency over the control group in bone marrow.

### 3.3 Mutation frequency in genomic DNA

As was the case with the TGR assay, the mutation frequencies generated by DS indicated that the esophagus had the highest increase in genomic mutation frequency among all NMPA-treated groups. The mutation frequencies were 48.78, 49.45, and 45.35 × 10^−8^ in the 5, 10, and 20 mg/kg NMPA-treated groups, respectively, compared to 6.55 × 10^−8^ in the vehicle control group (Figure 2B). These correspond to 7.4-, 7.5-, and 6.9-fold increases relative to the vehicle control. B[a]P-treated rats exhibited the smallest increase in mutation frequency in the esophagus, with a value of 15.14 × 10^−8^, corresponding to a 2.3-fold increase compared to the vehicle group.

In the liver, mutation frequencies for the 5, 10, and 20 mg/kg NMPA-treated groups were 11.14, 16.77, and 17.19 × 10^−8^, respectively. The fold changes were 1.6, 2.4, and 2.4-fold, respectively, compared to the vehicle group. The B[a]P-treated group showed a relatively large increase in liver mutation frequency, reaching 37.98 × 10^−8^, which represents a 5.4-fold increase compared to the vehicle control.

Finally, the bone marrow showed the least change in NMPA-induced mutation frequency as assessed by DS. The mutation frequencies in the NMPA-treated groups were 9.21, 9.48, and 8.7 × 10^−8^, with corresponding fold changes of 1.5-, 1.6-, and 1.4-fold, respectively, compared to the vehicle group. B[a]P treatment resulted in the highest increase in mutation frequency, reaching 39.67 × 10^−8^, representing a 6.6-fold increase relative to the vehicle control.

### 3.4 Mutation spectra

The mutation profiles in the liver, esophagus, and bone marrow of rats treated with NMPA, as well as those in the negative and positive control groups, were evaluated using the data generated by DS. A significant increase in mutation frequency was observed in the esophagus following NMPA treatment, with notable elevations across all six types of base substitutions, including T → A, T → C, T → G, and C → G, compared to the vehicle control group (Figure 3A). The mutational spectra for the NMPA-treated groups were significantly different from those of the vehicle controls (*p* < 0.0001). Unlike other small-molecule nitrosamines such as NDEA, which predominantly induce A:T → T:A transversions and A:T → G:C transitions (accounting for over 65% of total mutations), NMPA induced relatively uniform increases across all six possible types of base substitution. Moreover, no dominant pattern was identified in the trinucleotide mutation context following NMPA treatment, indicating a broadly distributed elevation across all trinucleotide contexts (Figure 3B).

**FIGURE 3:**
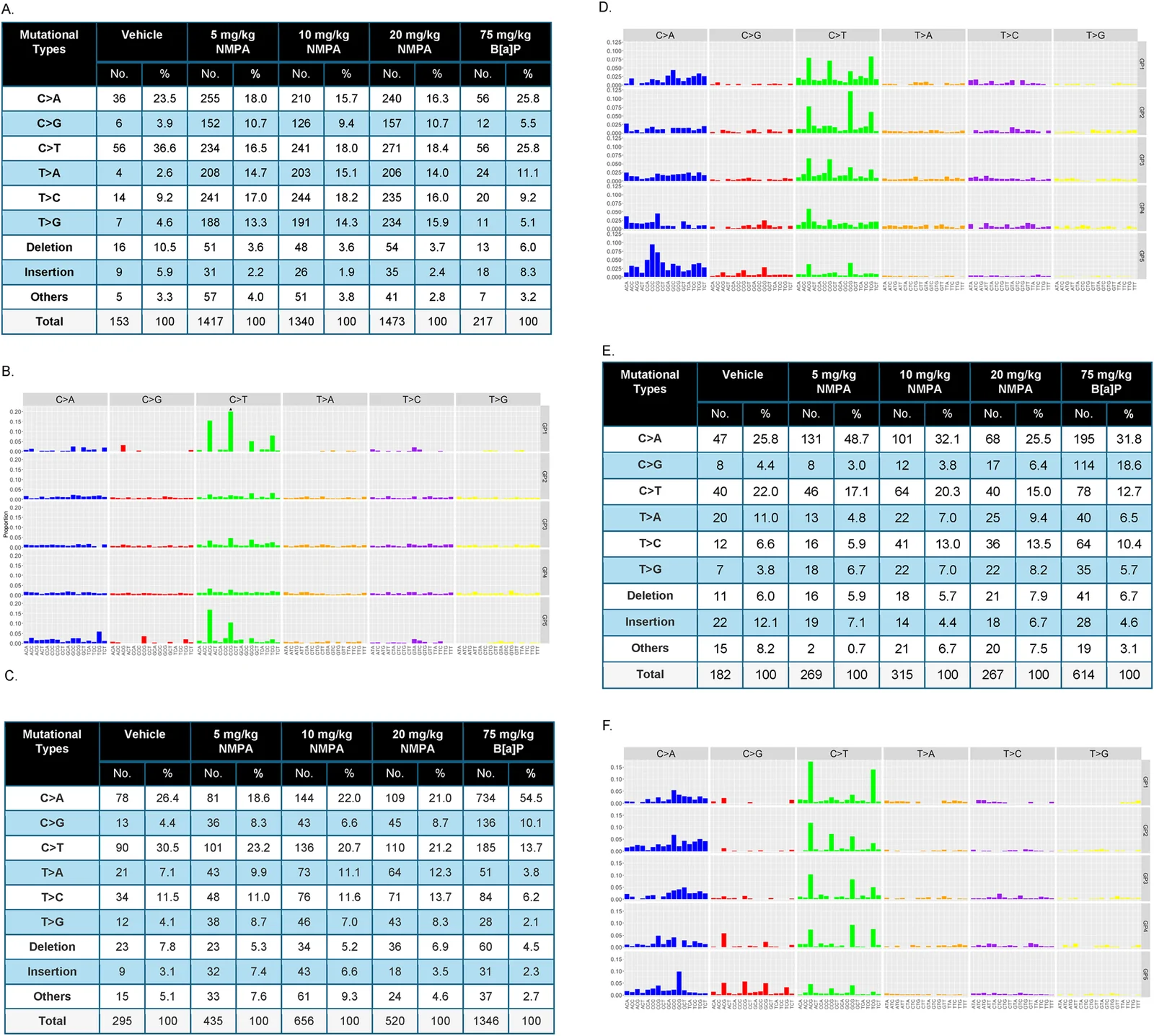
Mutation profile and trinucleotide spectra in the esophagus, liver, and bone marrow of the Big Blue rats treated with vehicle, *N*-nitroso-methylphenylamine (NMPA), or benzo[*a*]pyrene (B[*a*]P). Mutation profiles for the esophagus **(A)**, liver **(C)**, and bone marrow **(E)** are shown in the left panels. Corresponding trinucleotide spectra of single base substitutions for the vehicle control (GP1), NMPA (5 mg/kg - GP2; 10 mg/kg - GP3; and 20 mg/kg - GP4), and B[a]P (GP5) groups in the esophagus **(B)**, liver **(D)**, and bone marrow **(F)** are shown in the right panels. Mutations are shown in the 96-trinucleotide format, categorized by base substitution types (C→A, C→G, C→T, T→A, T→C, T→G) and the immediate 5′ and 3′ flanking nucleotides.

The B[a]P-treated group showed a slight increase in C → A transversions in esophageal samples, but its mutational spectrum was not significantly different from the vehicle control (*p* = 0.41).

The liver exhibited fewer mutations following NMPA treatment, with minimal differences in the mutation spectrum between the NMPA-treated and vehicle groups (*p* > 0.05; Figure 3C). However, B[*a*]P induced the greatest increase in C → A transversions, particularly in trinucleotide contexts such as CCA → CAA, CCC → CAC, and CCG → CAG in rat liver (*p* < 0.05; Figure 3D). This pattern is consistent with our previous studies on NEIPA, where similar elevations in C → A transversions were detected in liver for the B[a]P control (Xia et al., 2025).

NMPA treatment resulted in less than a 2-fold increase in mutation frequency in the bone marrow compared to the vehicle, and no significant differences were observed in the mutation spectra between these groups (*p* > 0.1; Figure 3E). C → A transversions remained the predominant B[a]P-induced mutations in the bone marrow as in the liver, with GCG → GAG and CCG → CAG being the most frequent trinucleotide contexts. Additionally, a significant increase in C → G transversions was observed in bone marrow, primarily at the trinucleotide contexts CCG → CGG, ACG → AGG, and GCG → GGG (*p* < 0.01; Figure 3F).

The TwinStrand DS rat mutagenesis panel evaluates mutations in 20 genomic regions across 18 chromosomes of the rat genome. Compared to the vehicle group, mutation frequencies were significantly elevated (*p* < 0.05) in all target regions in all NMPA treatment groups and tissues. The most pronounced increases in mutation frequency were observed in the esophagus (Figure 4A), while the bone marrow showed the smallest changes among the three tissues examined (Figure 4C). Moreover, mutation frequencies showed considerably less variation across the 20 genomic loci compared to observations from our previous study on NEIPA-induced mutations. No consistent differences were observed between genic and non-genic regions (Figures 4A–C).

**FIGURE 4:**
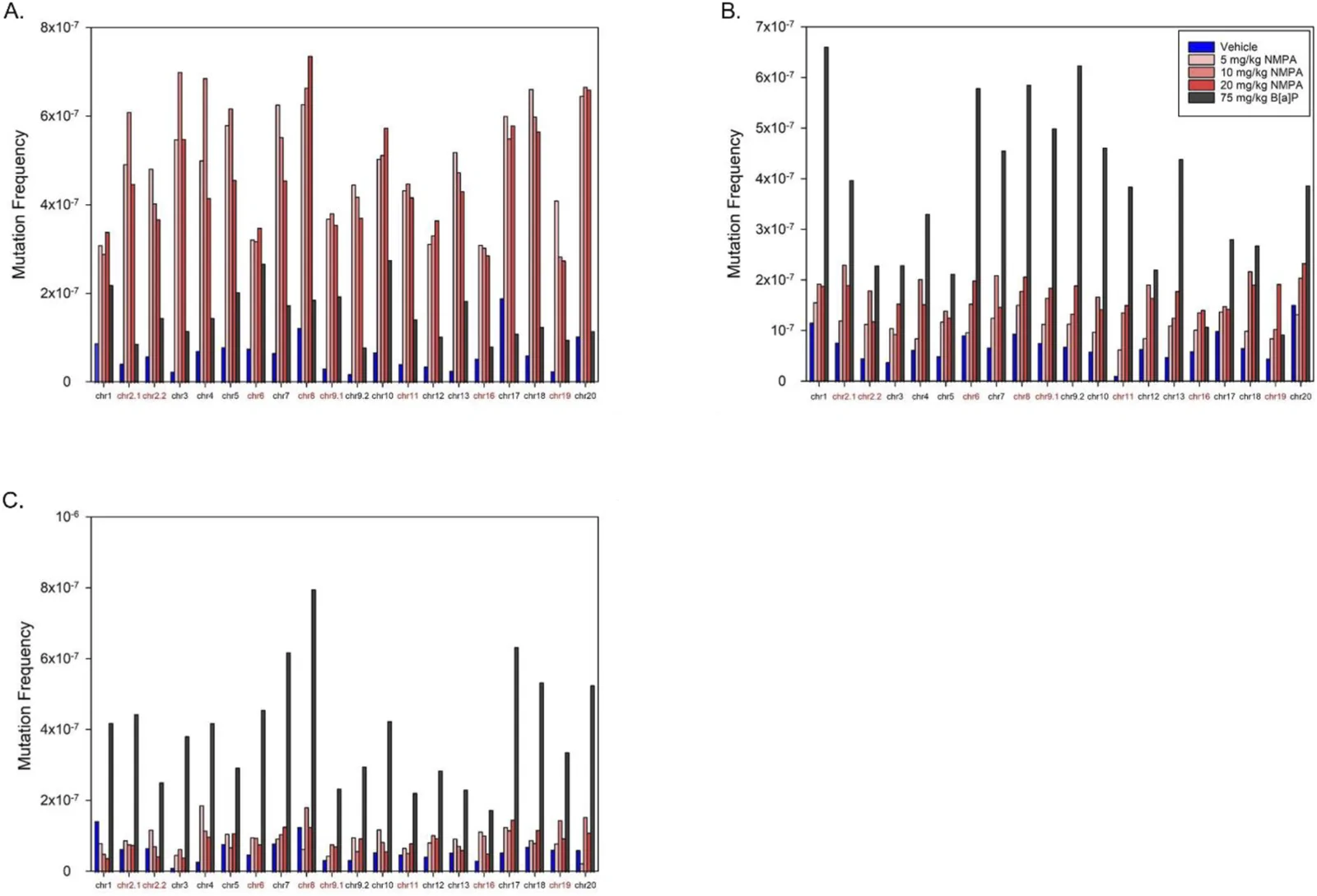
Mutation frequencies across 20 Duplex Sequencing target regions in esophagus **(A)**, liver **(B)**, and bone marrow **(C)** of the Big Blue rats treated with vehicle control, 5, 10, and 20 mg/kg *N*-nitroso-methylphenylamine (NMPA), or benzo[a]pyrene (B[*a*]P). All data are presented as mean. Bar colors in panels **(A)** and **(C)** correspond to the legend shown in panel **(B)**. Genic target regions are labeled in red, while the intergenic regions are labeled in black.

To explore whether the mutational signatures induced by NMPA treatment offer insights into potential carcinogenic mechanisms, we calculated the cosine similarity between the NMPA-induced signature in the esophagus and the signatures in the COSMIC single-base-substitution (SBS) signatures for genome rn6 (v3.3 - June 2022, https://cancer.sanger.ac.uk/signatures/downloads/). This database includes mutational signatures from 2,780 whole-genome variant calls produced by the ICGC/TCGA Pan Cancer Analysis of Whole Genomes (PCAWG) Network. The trinucleotide spectra from animals treated with the highest dose of NMPA (Group 4) showed the most similarity to SBS3, a signature proposed as a predictor of defective homologous recombination-based repair. For both the middle (Group 3) and high (Group 4) NMPA doses, SBS87, SBS40 and SBS9 also emerged as closely matched to the NMPA signature (Figure 5).

**FIGURE 5:**
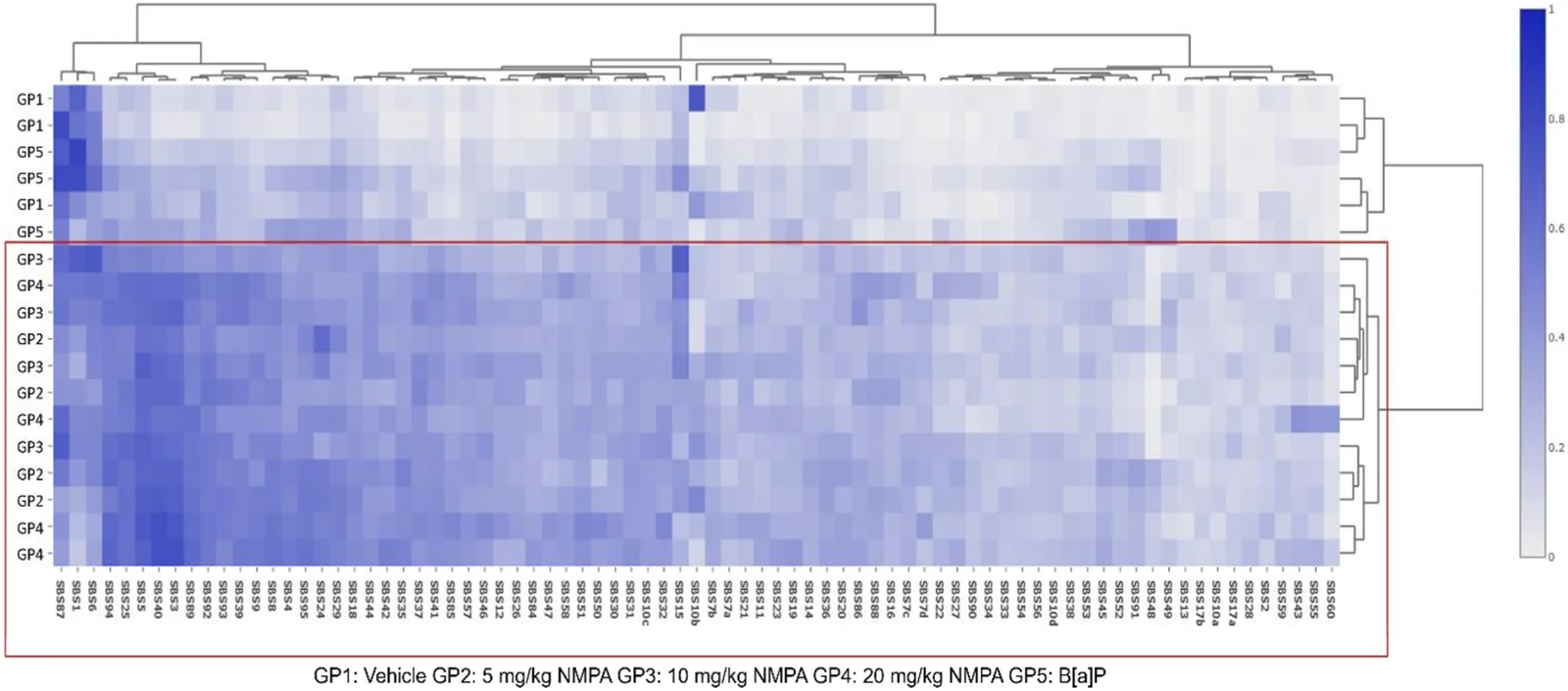
Cosine similarity between the trinucleotide spectra of each sample and the COSMIC single-base substitution (SBS) signatures (Catalogue Of Somatic Mutations In Cancer; genome rn6 (v3.3 - June 2022). SBS signatures were obtained from the COSMIC database (https://cancer.sanger.ac.uk/signatures/downloads/). The analysis was performed using TwinStrand Duplex Sequencing Mutagenesis Report software. The rats were treated with different doses of *N*-nitroso-methylphenylamine (NMPA), vehicle or benzo[*a*]pyrene (B[*a*]P); specific group details are provided at the bottom of the figure.

## 4 Discussion

Unlike other potent small-molecule nitrosamines such as NDEA and NDMA, which primarily target the liver, NMPA primarily induces tumors in the esophagus (Boyland, et al., 1964; Druckrey, et al., 1967; Goodall, et al., 1970; Kroeger-Koepke, et al., 1983; Schmähl, 1975). In this study, the esophagus exhibited the highest mutant/mutation frequencies and fold changes following NMPA exposure, indicating that the esophagus is significantly more sensitive than the liver or bone marrow for mutation induction. The high mutation burden in the esophagus suggests that NMPA may undergo a distribution and metabolic activation that differs from other nitrosamines. The mutation frequencies in bone marrow remained relatively unaffected, suggesting that bone marrow is not a suitable surrogate tissue for detecting NMPA-induced mutagenesis. This limited response in bone marrow may be attributed to its low metabolic capacity for NMPA activation, and that mutagenic metabolites of NMPA produced in other tissues may be short-lived and not effective via systemic circulation in producing mutation in bone marrow.

Given that NMPA was administered orally through drinking water, the esophagus is directly exposed during ingestion, resulting in a significantly higher local concentration of NMPA compared to internal organs such as the liver. This may lead to early, localized metabolic activation and DNA damage before systemic distribution. A previous tumorigenesis study showed that NMPA treatment via gavage, although resulting in tumors, was a much less effective route of administration than drinking water for inducing esophageal tumors (Goodall, et al., 1970). However, although the route of administration may partially account for the tissue specificity of NMPA, it does not explain why i.v. injection or gavage still induced tumors mainly in the esophagus and not in the liver.

Previous studies have demonstrated that NMPA is efficiently activated by CYPs (Snodin, et al., 2024; Stiborová et al., 1996). This metabolic activation leads to the formation of an unstable α-hydroxy intermediate, which decomposes through diazohydroxide formation, ultimately generating the highly electrophilic BDI that can bind to DNA to form DNA adducts (Koepke et al., 1990). The esophagus contains specific isozymes, such as CYP2A3 in rats, that are highly efficient at dealkylating asymmetric nitrosamines like NMPA. Thus, esophagus possesses the specific α-hydroxylation activity needed to convert NMPA into a reactive mutagen; also, compared with liver, it may have lower catalytic activity for simpler nitrosamines like NDMA. Esophageal microsomes have been shown to demethylate complex, asymmetric nitrosamines up to 20 times more rapidly than simpler ones like NDMA (Huang et al., 1992; Visoni et al., 2008). Thus, tissue-specific enzyme activity could be an important contributing factor to the tissue-specific mutagenicity of NMPA.

In addition, unlike NDMA, which is almost entirely cleared by the liver (the “first-pass effect”), NMPA largely bypasses hepatic metabolism and circulates to the esophageal mucosa, where it is then locally activated, potentially resulting in mutagenesis and carcinogenesis (Pylypiw et al., 1984). Furthermore, metabolites of NMPA in the liver can be efficiently removed by Phase I and II metabolism via denitrosation and conjugation by glucuronides, sulfonates, and glutathione. The esophagus, however, has limited detoxification capacity, potentially resulting in reduced clearance of highly reactive intermediates like BDI and increased local accumulation of DNA-damaging species (Burak et al., 1991; Carlson, 2019; Cross and Ponting, 2021; Ji and Schüürmann, 2012; Wade et al., 1987). Thus, the elevated mutation frequencies detected in the esophagus in this study are consistent with a greater vulnerability of this tissue to NMPA-induced genotoxicity due to its relatively low levels of detoxification.

The esophagus also lacks the robust repair mechanisms found in the liver, especially with regards to the activity of alkylguanine-DNA-alkyltransferase (AGT), an enzyme that repairs alkylguanine adducts. The very low level of this protective enzyme in the esophagus makes it far more vulnerable than the liver to the mutations induced by NMPA (Pegg, 1980; EFSA Panel on Contaminants in the Food Chain (EFSA CONTAM Panel), 2023). Although alkylating agents can induce DNA adducts at all the *O*- and *N*-atoms of nucleotides, *O*-alkylations are highly mutagenic and cytotoxic (Kaina et al., 2007). *O* ^6^-Alkylguanines are a critical type of DNA alkylation product, leading to mutations at G:C sites; *O* ^4^-alkylthymines are also mutagenic, causing mutations at A:T sites (Beranek, 1990; Zak et al., 1994). AGT can efficiently remove the DNA damage leading to mutations at G:C sites, which results in a high frequency of mutations in the A:T sites in the liver as compared with the esophagus (Samson et al., 1997; Sassanfar et al., 1991; Zak, et al., 1994). Our mutation profiles in the liver and esophagus were consistent with this pattern (Table 1): mutation induction by NMPA in the liver only resulted from mutations at A:T sites, while the esophagus induction involved increases in all six types of base substitution.

**TABLE 1:** Comparison of mutation frequencies across different types of mutations in the liver and esophagus of Big Blue rats treated with 5 mg/kg *N*-nitroso-methylphenylamine (NMPA) versus the vehicle control.

Types of mutations	Liver			Esophagus		
	Control (x10^−8^)	NMPA (x10^−8^)	Fold change	Control (x10^−8^)	NMPA (x10^−8^)	Fold change
G:C → C: G G:C → A:T G:C → T:A	4.45	5.04	1.1	4.01	24.44^***^	6.1
A:T → T:A A:T → G:C A:T → C:G	1.14	5.01^*^	4.4	1.49	14.39^***^	9.7
Indels and others	1.37	1.09	0.8	1.05	9.95^***^	9.5

* *p* < 0.05.

*** *p* < 0.001.

The NMPA doses used in this study were selected based on previous tumorigenicity studies (Lijinsky et al., 1983; Schmähl, 1975). Increased mutation frequencies in the esophagus were observed at all doses; however, a clear dose-response relationship was not seen for mutagenicity studies conducted using either the TGR transgene or by DS–all NMPA-treated animals had similarly elevated mutant frequencies in the esophagus. This was unfortunate as it limited our ability to compare the sensitivities of the two mutation analysis platforms. There also were no dose-response tumorigenicity data available for direct comparison; however, a high dose treatment by gavage resulted in a lower induction of tumors than treatments conducted via drinking water (Kroeger-Koepke, et al., 1983).

Although the mutant frequencies in the three NMPA-treated groups were similar, there were some potentially interesting trends in the responses: the middle dose group (10 mg/kg) exhibited slightly higher mutant frequencies than the lowest dose, whereas the highest dose group (20 mg/kg) showed a reduction in mutant frequency compared to the middle as well as lowest dose group. One possibility is that cytotoxicity might play a role in the high dose reduction because it can trigger apoptosis, thereby eliminating damaged cells before they can undergo DNA replication and mutation, ultimately resulting in lower observed mutant frequencies (Doak et al., 2007; Kirkland et al., 2005). In addition, high-dose NMPA may suppress cell proliferation. A cytostatic effect at higher dose levels has been observed in other genotoxicity studies–reduced rates of cell division can limit the consequent mutation fixation and detection of mutations because DNA damage cannot be converted to mutations without DNA replication (Bielas and Heddle, 2000). This requirement for DNA replication to fix mutations may explain the reduced mutant frequencies with 20 mg/kg NMPA despite potentially higher initial levels of DNA damage.

Utilization of both the TGR assay and DS in this study allows for a comparison of the relative performance of the two assays in detecting NMPA-induced mutagenesis and tissue-specific responses. DS detected NMPA-induced mutations *in vivo* and demonstrated higher sensitivity compared to TGR mutation assays, identifying mutagenicity that could not be measured by the TGR assay. In the esophagus, the DS Rat Mutagenesis panel detected 7.4-, 7.5-, and 6.9-fold increases in mutation frequency following 5, 10, and 20 mg/kg NMPA treatment, respectively, compared to vehicle control. In contrast, the corresponding *cII* assays found 4.7-, 5.6-, and 4.3-fold increases at the same doses. Similarly, in the liver, DS detected 1.6- to 2.4-fold increases across NMPA dose groups, while the *cII* assay reported slightly lower fold changes of 1.6- to 1.9-fold. In bone marrow, mutant frequencies were not significantly increased in the *cII* assay (1.0–1.7-fold changes) but mutation frequencies were significantly increased in the NMPA-treated rats (1.4–1.6-fold) when measured by DS. The data highlights DS’s higher sensitivity to NMPA-induced mutagenesis relative to the TGR assay, consistent with our previous study with NEIPA (Xia, et al., 2025). These findings support the enhanced precision and sensitivity of DS, particularly in tissues with modest or variable mutagenic responses.

NMPA falls into the Carcinogenic Potency Categorization Approach (CPCA) Potency Category (PC) of PC2. PC2 ranking indicates that, structurally, NMPA is anticipated to be a moderately potent carcinogen, and PC2 drug impurities are accorded a relatively low Acceptable Intake (AI) limit of 100 ng/day (Kruhlak et al., 2024)). Comparison of this predicted potency and assigned AI limit with AI limits derived from empirical cancer data for NDMA and NDEA indicate the carcinogenic potency of NMPA is nearly as great as NDMA and NDEA, which have AI limits of 96 and 26.5 ng/day (FDA, 2025).

In addition, cosine similarity analysis comparing the trinucleotide mutation spectra of the samples in this study and the COSMIC SBS signatures for genome rn6 provide insights into the potential carcinogenicity of NMPA. The esophageal mutation signature from the highest NMPA dose group had the greatest similarity to SBS3, a signature frequently observed in cancer samples. This signature is associated with defective homologous recombination-based DNA repair. This finding suggests that NMPA-induced damage may overwhelm or inhibit this repair pathway in the esophagus, contributing to its high mutagenicity. At both the middle and high NMPA doses, strong correlations also were observed between the NMPA signature and SBS87, SBS40 and SBS9. SBS87 has been identified in relapsed acute lymphoblastic leukemia patients, while SBS9 is commonly found in chronic lymphocytic leukemia, particularly in cases with immunoglobulin gene hypermutation (IGHV-mutated). SBS9 is thought to reflect error-prone translesion DNA synthesis during somatic hypermutation. Although SBS9 is classically linked to lymphoid malignancies, its detection in non-lymphoid tissue such as the esophagus may indicate activation of similar error-prone DNA repair or translesion synthesis pathways in response to NMPA-induced genotoxic stress. SBS40 is of unknown etiology but has been observed in various cancers. Its presence could suggest a currently uncharacterized mechanism of NMPA-induced damage or repair.

As indicated in the ICH M7 (R1) guidance (ICH, 2017), mutagenicity is recognized as a surrogate endpoint for the identification of carcinogenic drug impurities. Currently efforts are underway to use TGR (and possibly ECS) mutagenesis data quantitatively to set limits for nitrosamine drug impurities with no compound-specific cancer data and no cancer data suitable for read-across estimates of carcinogenic potency. The presence of cancer-related mutational signatures in our study is consistent with the utility of mutagenesis data as a predictive tool for assessing the carcinogenic potential of NMPA. Importantly, the tissue-specific mutagenicity observed in this study was consistent with available carcinogenicity data. The esophagus, which exhibited the highest mutation burden, also showed the highest tumor incidence (17/20 rats), whereas the liver showed a small to moderate increase in mutation and only 1/20 rats developed liver tumors. Bone marrow showed minimal mutagenic changes and no tumor development (0/48). These findings are summarized in Table 2.

**TABLE 2:** Summary of tissue-specific mutagenicity and carcinogenicity in rats following *N*-nitroso-methylphenylamine (NMPA) exposure.

Tissue	Mutagenicity (TGR *cII* assay)	Mutagenicity (duplex sequencing)	(Tumors/total rats)
Esophagus	High increase	Highest increase	17/20
Liver	Small increase	Moderate increase	1/20
Bone marrow	No significant change	Slight increase	0/48

Tumor incidence is expressed as the number of animals with tumors relative to the total number of animals evaluated for each tissue (Lhasa Carcinogenicity Database; Study ID: 44605646).

However, this correspondence between target tissues for carcinogenesis and mutagenesis raises the issue of tissue selection for mutagenesis analysis in the absence of carcinogenicity data. It is clear from the over 100 cancer bioassays performed on nitrosamines that liver is often the target tissue for carcinogenesis, but it is not the only tissue that can be a target. For instance, in the case of NMPA, it is the esophagus (Lijinsky et al., 1982); while in the case of *N*-butyl-*N*-(4-hydroxybutyl)nitrosamine, it is the bladder (Vasconcelos-Nobrega et al., 2012). Over 10 tissues have been identified as targets for nitrosamine carcinogenesis in rats and mice (Lhasa, 2026). When mutagenesis data are used quantitively for setting AI limits, the cancer targets will not be known and the selection of tissues for mutation analysis must be carefully justified.

## 5 Conclusion

This study provides evidence for the tissue-specific mutagenicity of NMPA, with the esophagus as the primary target tissue. Both the *cII* transgenic assay and DS identified significant increases in mutation frequency in the esophagus. NMPA treatment resulted in increases in all types of base pair substitutions. The esophagus has been demonstrated as the target tissue for tumor development following NMPA exposure in rats, and the mutagenicity assay results in our study align with findings from carcinogenicity studies. Moreover, the presence of cancer-related mutational signatures further supports the strong carcinogenic potential of NMPA in esophageal tissue. This tissue specificity may arise from the specific chemical structure of NMPA, localized exposure, low first-pass hepatic metabolism, esophagus-specific metabolic activation, and low DNA repair capacity in the esophagus. Additionally, our results highlight the ability of DS to detect subtle mutagenic responses and the need to scientifically justify tissue selection for nitrosamine mutagenicity testing, particularly when evaluations are restricted solely to the liver.

## Data Availability

The original contributions presented in the study are included in the article/supplementary material, further inquiries can be directed to the corresponding author.
